# The complete chloroplast genome of *Vancouveria planipetala*, with implication for the phylogeny of Ranunculales

**DOI:** 10.1080/23802359.2018.1473726

**Published:** 2018-05-21

**Authors:** Zhenyu Jin, Jocelyn M. Losh, Wenqing Ye, Pan Li

**Affiliations:** Key Laboratory of Conservation Biology for Endangered Wildlife of the Ministry of Education, and Laboratory of Systematic & Evolutionary Botany and Biodiversity, College of Life Sciences, Zhejiang University, Hangzhou, China

**Keywords:** Basal group, Berberidaceae, Epimedium, Eupteleaceae, Papaveraceae

## Abstract

*Vancouveria planipetala* (Berberidaceae) is a perennial herb which has high ornamental and ecological values. In this study, we assembled the complete chloroplast (cp) genome of *V. planipetala*. The whole cp genome of *V. planipetala* is 156,871 bp in length, comprising a pair of inverted repeat (IR) regions (25,888 bp) separated by a large single copy (LSC) region (88,321 bp) and a small single copy (SSC) region (16,772 bp). The cp genome contains 114 unique genes, including 80 protein-coding genes, 30 tRNA, and four rRNA genes, with 17 genes duplicated in IRs. Phylogenetic analyses showed that Papaveraceae is the basal group of Ranunculales.

*Vancouveria planipetala* Calloni (Berberidaceae) is a perennial herb occurs in northwestern California and southwestern Oregon (Whetstone et al. 1997). The lovely dropping white flowers of *V. planipetala* make it a nice ornamental plant. By now, genetic resources of *V. planipetala* are very limited. In this study, we sequenced the chloroplast (cp) genome of *V. planipetala* and analysed its phylogenetic position in Ranunculales.

The leaf materials of *Vancouveria planipetala* were collected from a cultivated plant in Far Reaches Farm (Port Townsend, WA). Voucher specimen (LP162863) was deposited in the Herbarium of Zhejiang University (HZU). The total DNA was extracted using a modified CTAB method (Doyle and Doyle 1987) and then sequenced using Illumina HiSeq^TM^ 2000 (San Diego, CA). The raw data were filtered and assembled using CLC Genomics Workbench 8 (Qiagen, Valencia, CA). Then, all the contigs were aligned to the reference cp genome of *Epimedium lishihchenii* (GenBank accession number: NC_029944.1; Zhang et al. 2016) using BLAST (NCBI BLAST v2.2.31) search and the draft cp genome of *V. planipetala* was constructed by connecting overlapping terminal sequences. Finally, the whole sequence was annotated with Geneious 11.0.2 (Biomatters Ltd., Auckland, New Zealand). The complete cpDNA sequence of *V. planipetala* has been submitted to GenBank (MH337373). Maximum likelihood (ML) analysis was implemented in RAxML-HPC v8.2.10 on the CIPRES Science Gateway V. 3.3 (Miller et al. 2010) and Bayesian inference (BI) analysis was performed in MrBayes v3.2.6 (Ronquist and Huelsenbeck 2003), with *Sabia yunnanensis* (NC_029431.1; Sun et al. 2016) as the outgroup.

The whole cp genome of *Vancouveria planipetala* is 156,871 bp in length, which contains a typical quadripartite structure of a large single copy region (LSC: 88,323 bp) and a small single copy region (SSC: 16,772 bp) separated by a pair of inverted repeat regions (IRs: 25,888 bp). The total GC content of the complete cp genome, LSC, SSC, IR regions is 39.0%, 37.6%, 32.8%, 43.2%, respectively. The genome contains 114 unique genes, including 80 protein-coding genes, 30 tRNA genes, and four rRNA genes. Seventeen genes were duplicated in the IR regions. There are 15 genes (six tRNA genes and nine protein-coding genes) with one intron and three genes (rps12, clpP, and ycf3) with two introns. The rps12 gene was trans-spliced, with the 5′ end located in the LSC region and the 3′ end duplicated in the IR region.

Taking advantage of publicly available cp genomes, we reconstructed the phylogenetic relationship of Ranunculales. The tree topologies yielded by ML and BI analyses were consistent with each other (Figure 1). The phylogeny of Ranunculales was successfully resolved, with full support [ML bootstrap (BS) = 100%, BI posterior probability (PP) = 1] at almost all nodes. Papaveraceae was revealed to be the basal group of Ranunculales, which conflicts with previous studies that found Eupteleaceae to be the first diverging clade (Wang et al. 2009; Sun et al. 2017), and thus more research is needed to confirm the basal group of Ranunculales. Within Berberidaceae, *Vancouveria planipetala* and *Epimedium lishihchenii* were sisters.

**Figure 1.**
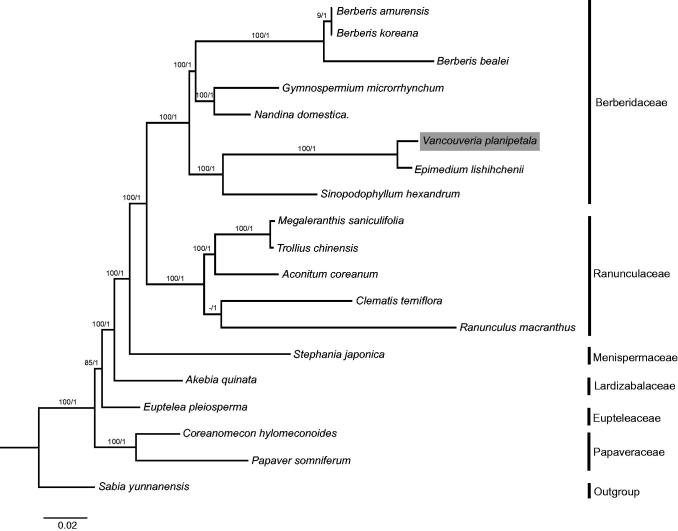
Molecular phylogeny of Ranunculales based on 17 complete cp genomes. The accession numbers are listed as below: *Berberis amurensis* (NC_030062.1); *B. koreana* (NC_030063.1); *B. bealei* (NC_022457.1); *Gymnospermium microrrhynchum* (NC_030061.1); *Nandina domestica* (NC_008336.1); *Vancouveria planipetala* (MH337373); *Epimedium lishihchenii* (NC_029944.1); *Sinopodophyllum hexandrum* (NC_027732.1); *Megaleranthis saniculifolia* (NC_012615.1); *Trollius chinensis* (NC_031849.1); *Aconitum coreanum* (NC_031421.1); *Clematis terniflora* (NC_028000.1); *Ranunculus macranthus* (NC_008796.1); *Stephania japonica* (NC_029432.1); *Akebia quinata* (NC_033913.1); *Euptelea pleiosperma* (NC_029429.1); *Coreanomecon hylomeconoides* (NC_031446.1); *Papaver somniferum* (NC_029434.1); *Sabia yunnanensis* (NC_029431.1).
